# Patient Positioning and Treatment Techniques in Total Skin Irradiation: A Scoping Review

**DOI:** 10.3390/cancers17081276

**Published:** 2025-04-09

**Authors:** Andrea Lastrucci, Emanuele Canzani, Neda Haghighatjou, Livia Marrazzo, Nicola Iosca, Yannick Wandael, Daniele Giansanti, Renzo Ricci, Monica Mangoni, Stefania Pallotta, Gabriele Simontacchi, Lorenzo Livi

**Affiliations:** 1 Department of Allied Health Professions, Azienda Ospedaliero-Universitaria Careggi, 50134 Florence, Italy; 2 Radiation Oncology Unit, Azienda Ospedaliero-Universitaria Careggi, 50134 Florence, Italy; 3 University of Florence, 50121 Florence, Italy; 4 Medical Physics Unit, Careggi University Hospital, 50134 Florence, Italy; livia.marrazzo@unifi.it (L.M.); stefania.pallotta@unifi.it (S.P.); 5 Department of Experimental and Clinical Biomedical Sciences “M. Serio”, University of Florence, 50134 Florence, Italy; 6 Centre TISP, Istituto Superiore di Sanità, 00161 Rome, Italy; daniele.giansanti@iss.it

**Keywords:** total skin electron irradiation, patient positioning, radiotherapy

## Abstract

**Introduction**: Total skin irradiation (TSI) is a radiotherapy technique used to treat cutaneous lymphomas, such as mycosis fungoides. A key aspect of the success of the treatment is the correct positioning of the patient, which ensures a homogeneous distribution of radiation over the skin surface, minimizing exposure to the surrounding healthy tissues. **Materials and Methods**: Following the preferred reporting items for systematic review and meta-analysis (PRISMA) extension for scoping reviews and the Arksey and O’Malley framework, electronic searches of EMBASE, PubMed, SCOPUS, and the Web of Science were conducted to identify original studies detailing positioning techniques for TSI in clinical practice. **Results**: A total of 44 studies were included, reporting a range of positioning techniques used in TSI. The selected articles were divided into four categories: Stanford, rotational, tomotherapy, and mixed (including studies comparing two or more of the previously mentioned techniques). Articles concerning the Stanford technique were predominant (*n* = 33; 75.0%), followed by those addressing the rotational technique (*n* = 5; 11.4%), tomotherapy (*n* = 3; 6.8%), and mixed (*n* = 3; 6.8%). Studies on the Stanford technique described both the original method and its modifications, including variations such as the lying position, and analyzed its strengths and limitations. Research on the rotational method examined different device configurations and implementation strategies to optimize dose distribution and treatment efficiency. Mixed articles compared the Stanford and rotational techniques, highlighting their similar dose uniformity while examining differences in treatment efficiency and practical implementation. **Conclusions**: Despite its widespread use, the Stanford technique is associated with challenges that have led to the exploration of alternative positioning strategies. The rotational technique addresses some of these limitations, while tomotherapy offers advanced immobilization but raises toxicity concerns. Future research should focus on optimizing the balance between efficacy, safety, and practicality of treatment in clinical settings.

## Simple Summary

Total skin irradiation (TSI) is a radiotherapy technique used to treat cutaneous lymphomas like mycosis fungoides. Correct patient positioning is crucial for homogenizing the dose across the skin surface area while minimizing exposure to healthy tissues. This scoping review included 44 studies on TSI positioning techniques from 1982 to 2024, categorizing them into Stanford, rotational, tomotherapy, and mixed approaches. The Stanford technique was the most common (75.0%), followed by rotational (11.4%), tomotherapy (6.8%), and mixed (6.8%) techniques. While the Stanford technique is widely used, it has drawbacks like inconsistent dose distribution and long treatment durations. The rotational method improves efficiency by reducing session times, whereas tomotherapy provides better organ protection and immobilization but has limitations like prolonged treatment sessions and higher doses delivered to deep organs. Despite the dominance of the Stanford technique, ongoing challenges have driven the exploration of alternative methods to optimize TSI treatment outcomes.

## 1. Introduction

Total skin irradiation (TSI) is a radiotherapy technique that has been used since the 1950s, primarily for the treatment of cutaneous T-cell lymphoma, a chronic and progressive form of lymphoma known as mycosis fungoides [1,2,3,4,5]. The primary goal of TSI is to deliver irradiation to the patient’s entire skin at a precise depth, ensuring a uniform dose across the target volume while minimizing toxicity [4,6]. An important aspect of TSI is the positioning technique, which ensures setup reproducibility and provides maximum comfort for the patient during the procedure [6]. The positioning method should ensure that the maximum skin surface area receives irradiation; any areas of the skin receiving less than 80% of the prescribed dose during electron treatment should be supplemented with additional irradiation [2,6,7,8,9]. To successfully implement TSI in clinical practice, a strong collaboration among a dedicated multidisciplinary team—including physicists, radiation therapists, and radiation oncologists—is essential [2,10]. In clinical practice, various TSI delivery techniques have been developed worldwide over the years to address the challenging task of achieving homogeneous skin irradiation. These techniques take into account technological requirements, geometric conditions, patient alignment, the number of treatment fields, and the dose fractionation schemes specific to each radiotherapy center [6,11]. Globally, the most commonly used techniques for TSI delivery are the Stanford technique, the rotational technique, and more recently, tomotherapy.

The original Stanford technique, described in the study by Kazmark et al., featured four body orientations—anterior, posterior, and lateral fields—with the patient in a standing position [12]. This approach was later modified by Page et al., who introduced a six-field technique for improved treatment setup [13]. Specifically, the Stanford technique involved treating patients in six standing positions (anterior, posterior, and four lateral oblique positions), at a specific distance from the gantry, and angled to ensure complete treatment field coverage with the electron beam. AAPM Report 23 served as the primary guideline for the implementation of this technique [3].

The rotational technique, developed at the McGill University Health Centre in the early 1980s, was addressed in five of the selected articles [14,15,16,17,18]. This technique involves rotating platforms or frames to ensure dose homogeneity, with patients either standing or lying in specific positions.

Tomotherapy enabled the delivery of intensity-modulated, rotational radiation therapy and offered several advantages, including a high degree of flexibility, improved dose optimization, and the ability to treat long and complex targets [19,20].

The study by Piotrowski et al. reviewed the techniques and positioning of patients; however, it was published in 2013. In recent years, technological advancements in radiotherapy—both in linac devices and immobilization devices—have further improved the positioning techniques for TSI.

The aim of this review is to evaluate the evolution of positioning techniques used in clinical practice for TSI over the years and to identify the strengths and weaknesses of each technique as reported in the literature. The research question guiding this review is as follows: What is the evidence on the evolution of positioning techniques over time and what are the main strengths and weaknesses of each? This review focuses on technical and procedural aspects rather than clinical outcomes.

## 2. Materials and Methods

An electronic literature search was conducted in accordance with the Preferred Reporting Items for Systematic Review and Meta-Analysis (PRISMA) extension for scoping reviews [21] and the Arksey and O’Malley framework [22]. The goal was to identify studies focusing on positioning techniques used in the delivery of TSI. Four major databases—Embase, PubMed, SCOPUS, and Web of Science (WOS)—were queried using search strings developed in collaboration with the University of Florence Library System (detailed in Supplementary Materials, Table S1). The search was completed on 27 June 2024.

Studies were deemed eligible if they met the following inclusion criteria:

- Articles were published in English.
- Studies addressed patient positioning techniques implemented in clinical practice during TSI.
- Studies involved human subjects or phantom models.
- Primary research studies were classified under established scientific research frameworks [23].

Exclusions included conference proceedings, congress abstracts, reports, non-English publications, secondary studies, and articles irrelevant to the research topic. This review was conducted in collaboration with the Department of Allied Health Professions and the Radiation Oncology Unit of Careggi University Hospital.

The references identified during the search were uploaded to Mendeley Reference Manager v2.120.1 for efficient bibliographic management. Duplicates were removed first using the software’s automated detection feature, followed by a manual review to ensure none were overlooked.

Study selection occurred in multiple stages. Initially, two independent reviewers (A.L. and E.C.) screened titles and abstracts to exclude irrelevant studies. Articles deemed potentially eligible were then subjected to full-text assessment based on predefined inclusion criteria.

All discrepancies were resolved through focused discussions between the independent reviewers with the involvement of a third reviewer (G.S.).

Data extraction was facilitated using a custom-designed electronic database that systematically monitored, updated, and exported collected information for analysis. Eligible articles underwent thorough review and critical evaluation to confirm their inclusion in the scoping review. An analytic approach was used for data analysis, specifically, the selected studies were synthesized and discussed by the research team and organized using a standardized data extraction form developed in Microsoft Excel (Redmond, Washington).

Key characteristics of each study, including delivery technique, patient position, and shielding used, were carefully extracted and reported. This synthesis was performed to identify all characteristics of each TSI technique in order to determine the strengths and weaknesses of each TSI technique, categorizing studies by authorship, publication year, country of origin, sample size, study title, shielding device usage, and the main technical features of the radiation treatment. No ethical approval was necessary for this study.

## 3. Results

### 3.1. Study Inclusion and Characteristics

**Figure 1:**
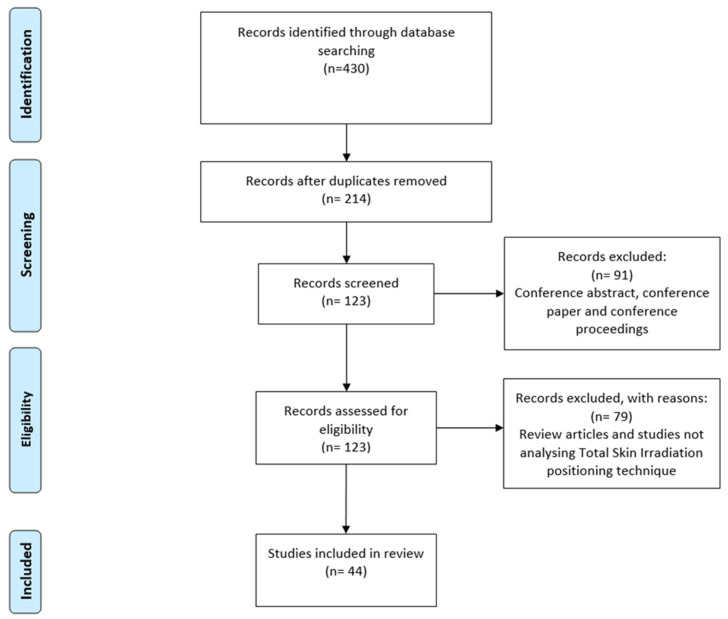
PRISMA flowchart of study selection.

Out of 430 articles, 44 met the predefined inclusion criteria (Figure 1). The main characteristics of the study samples are summarized in Figure 1, which presents the study selection process using a PRISMA diagram. 

The included articles were published between 1982 and 2022. Most studies focused on adult patients (*n* = 41; 93.2%), with only a few addressing pediatric patients (*n* = 3; 6.8%). Sample sizes varied widely, ranging from 1 to 85 in studies that reported this information (*n* = 18; 41%), while 26 studies (59%) did not specify sample size. The studies were conducted in 17 countries, with the majority originating from the United States (*n* = 19; 43.2%), followed by Canada (*n* = 4; 9.1%), Iran (*n* = 3; 6.8%), and Germany (*n* = 3; 6.8%).

**Figure 2:**
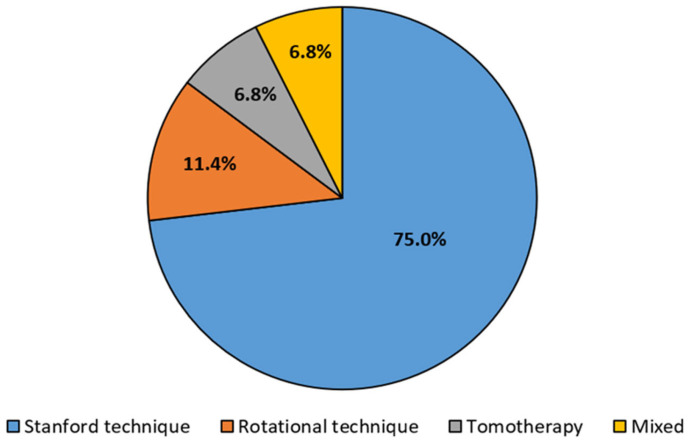
Retrieved articles are categorized based on the TSI technique used.

The articles analyzed various TSI patient positioning methodologies, categorized into four main techniques: the Stanford technique (*n* = 33; 75%), the rotational technique (*n* = 5; 11.4%), tomotherapy (*n* = 3; 6.8%), and mixed methodologies (*n* = 3; 6.8%), which compared two or more techniques. The Stanford category included studies employing the original Stanford technique and its modifications. This classification is illustrated in Figure 2.

An overview of the studies included is presented in Table 1.

### 3.2. Stanford Technique

Among the 44 included articles, the majority addressed the Stanford technique (*n* = 33; 75%) [10,24,25,26,27,28,29,30,31,32,33,34,35,36,37,38,40,41,42,43,44,45,46,48,50,53,54,55,57,59,60]. In 16 of these 33 studies (48.5%) [10,23,28,29,30,35,36,37,39,40,41,44,45,53,54,59], protective shielding for organs at risk was mentioned. Shielding often included thin layers of lead to protect nails and eyes. For example, Rahimy et al. [54] described a 3D-printed patient-specific scalp shield for hair preservation, while Shraiff et al. [60] reported lead shielding for testicle protection. Treatment source skin distances (SSDs) varied significantly, ranging from 124 cm [46] to 600 cm [54], influenced by factors such as bunker dimensions, patient size, and equipment utilized.

Three studies (10%) [11,30,32] focused on pediatric patients, all of whom were anesthetized during treatment. In the study by Bao et al. [35], a rotating harness system was used to position sedated pediatric patients, covering only the torso while allowing free movement of limbs. Constructed from a material minimizing electron beam attenuation, the harness was attached to a rotating plate with four belts and carabiners to facilitate patient rotation and enhance treatment precision. Earley et al. [30] modified the Stanford technique by positioning the pediatric patient close to the floor, using a treatment distance of 200 cm. Oblique positioning was achieved by rotating the gantry by 60 degrees and posterior beam delivery was conducted with the patient prone. Also, in the study by Kron et al. [46], a 2-year-old patient was treated in the supine position using a customized cradle with a thin window base and poly (methyl-methacrylate) (PMMA) frame.

Several studies reported deviations from the standard Stanford technique, either in beam delivery methods or in patient positioning during irradiation. While most studies (*n* = 19, 57.6%) treated patients in a standing position [24,26,28,29,32,33,34,35,36,41,42,43,44,48,50,54,57,59,60], others employed a modified Stanford technique treating adult patients in a supine position [25,27,30,31,37,38,40,46,56]. Some studies compared techniques with patients in both standing and reclined positions [10,45,52,53,55]. For example, Koken et al. [10] described TSI practices in the Netherlands and Belgium, noting that half of the institutions (two out of four) delivered TSI in the standing position with the patient in a standing position, while the other half used a stretcher for prone or supine treatment. The study by Ackerson et al. [55] proposed a dual approach to patient positioning, offering the option of either a standing or recumbent position, thereby enabling treatment for patients who might otherwise be ineligible for TSI.

The study by Gerbi et al. [25] proposed an arc irradiation technique with patients in a reclined position. This method utilized six patient positions and two symmetric ± 48° arc electron beams. The central axis of the first arc was positioned 95 cm above the junction point of the fields, while the axis of the second arc was placed 95 cm below the same point. Wu et al. [16] suggested an alternative to the traditional Stanford fields for non-ambulatory patients, employing vertical dual-field (VDF) and oblique junction field (OJF) techniques to ensure dose coverage. The VDF field replaced the AP and PA fields of the Stanford technique. A complete treatment cycle spanned two days, alternating between supine and prone positions to achieve full-body irradiation.

As reported in several articles, the boost was administered at the end of treatment using conventional electron beam fields to target underdosed areas, which were usually assessed for each patient. Underdosed areas typically included the top of the head, the under-surface of the pendulous breast, the perineal area, the upper medial aspect of the thighs, and the soles of the feet [15,24,25,26,27,29,31,32,33,35,36,41,43,45,46,56,59,60].

### 3.3. Rotational Technique

Five studies [14,15,16,17,18] employed the ‘dual-field’ technique, where the gantry was positioned laterally and adjusted to different angles relative to the horizontal axis for each beam. All articles provided comprehensive descriptions of patient setups, including devices used to facilitate rotation during treatment. For example, Kumar et al. [15] described patients positioned upright on a platform rotating at 5 rpm, with lead shielding to protect the eyes. The study by Wu et al. [16] described the use of three wooden frames—one static and two rotational—that supported patients in either supine or prone positions to ensure homogeneous dose distribution using a 9 MeV energy beam. The following six positions were used: three with the frame tilted forward and three tilted backward for prone, following the same arrangement supine. Patients were positioned with their arms raised above their heads, forming 90° angles relative to the torso, with fingers fully extended and legs spaced shoulder-width apart to maximize radiation exposure. Throughout treatment, the palms and face were maintained in the same orientation. The study by Hensley et al. [17] described a turntable system where patients, positioned upright, were stabilized by holding a bar. To mitigate underexposure of the inner thighs, patients adopted the ‘swordsman position’, alternating leg extension daily. The use of shielding to protect organs at risk during irradiation, specifically for the nails of the hands and feet, as well as the crystalline lens, was also mentioned in the article. Similarly, the study by Ding et al. [18] introduced a platform with a freely rotating hand-held bar anchored to the ceiling, which provided stabilization and ensured the reproducibility of the patient setup. The administration of the boost to underdosed skin areas was also described in the articles by Hensley et al. [17] and Ding et al. In contrast, the study by Wu et al. reported that the use of a special rotation technique made it possible to eliminate the need for boost fields on the vertex of the scalp and the soles of the feet, as required by the Stanford technique.

### 3.4. Tomotherapy

Over the years, the use of tomotherapy for TSI delivery has been increasingly documented in the literature. In this review, three of the selected articles [39,49,61] specifically focused on its application, examining its use across varying sample sizes. The study by Sarfehnia et al. [39] treated a single individual, the study by Haraldsson et al. [49] included two pediatric patients, and the study by Wang et al. [61] involved a cohort of six.

Regarding immobilization devices, the study by Sarfehnia et al. [39] treated a supine anesthetized pediatric patient immobilized with a Vac-Lok cushion and a thermoplastic head mask. The study by Haraldsson et al. [49] described the use of a large vacuum cushion (VacFix), an individually molded neck rest, and a five-point open-face thermoplastic mask, adding wetsuit socks, a hood, and gloves. Patients were positioned with arms and hands close to the trunk, knees slightly flexed, and feet immobilized with the vacuum cushion. Treatment was delivered while wearing a full neoprene suit as a bolus to ensure dose uniformity. Additionally, shields were used to protect the eyes, lips, and genitals. The study by Wang et al. [61] similarly reported the use of thermoplastic masks for the head, neck, thorax, and abdomen, while patients were immobilized in the supine position with their lower limbs secured in a vacuum cushion. These patients also wore a 5 mm neoprene suit.

Studies consistently highlight the advantages of MVCT imaging in ensuring accurate patient setup before each treatment session, enabling corrections to patient positioning prior to treatment delivery.

In the study by Sarfehnia et al. [39], radiochromic films were used to measure the skin dose during one, two, three, and seven fractions. The films were distributed over the patient’s body, with several pieces of film placed under the mask and between the patient’s skin and the Vac-Lok cushion. This was to determine whether there were hot or cold spots in regions where the high-density equipment was in contact with the skin. In the study by Haraldsson et al. [49], in vivo dosimetry was performed using at least 20 film strips, each measuring 1 × 1.5 cm^2^, taped at several positions on the patients’ skin. The bolus effect of the neoprene wet suit fitted on the phantom was quantified by paired film measurements in which a film was placed under the wetsuit for the first measurement and replaced without the wetsuit for the second measurement. In addition, a film strip was placed on a 20 cm thick fixed water plate and irradiated with and without a 200 × 200 × 7 mm^3^ square of neoprene to measure the build-up effect of the neoprene.

### 3.5. Mixed Techniques

Three articles [47,51,58] analyzed and compared at least two approaches. In all studies, the Stanford technique was compared with the rotational technique. In the study by Ding et al. [58], a comparison—in terms of skin dose distributions between the rotational and Stanford techniques—was conducted at three different SSDs (316 cm, 500 cm, and 700 cm). The findings obtained analyzing dose-volume histograms showed that both techniques provided very similar dose distributions to the patient’s skin with only small differences in some local areas for the patients treated. The study by Ansari et al. [47] compared the dose distribution between Stanford and rotational techniques, finding comparable homogeneity in transverse dose distribution when a degrader plane was used. The rotational technique, requiring a rotating plate (3 rpm) synchronized with the accelerator and adequate ventilation, was recommended for centers with lower dose rate accelerators due to its shorter treatment time compared to the Stanford method. The study by Falahati et al. [51] acknowledged the primary advantage of rotational techniques in reducing treatment time and reported similar findings regarding dose uniformity between the two methods. However, they recommended the stationary technique, citing challenges with the rotational approach, including concerns about patient comfort, safety, and the risk of non-uniform platform rotation.

## 4. Discussion

This scoping review analyzed the positioning techniques in TSI and identified 44 relevant studies out of 430 articles initially screened. Despite being developed over 60 years ago [12], the Stanford technique emerged as the most frequently analyzed, accounting for 75.0% (*n* = 33) of the studies. The literature extensively examines its clinical applications, effectiveness, and limitations. The Stanford technique involves irradiating the patient’s entire skin in six positions. Over the years, this technique has undergone modifications and adaptations to suit different clinical settings and to improve the homogeneity of the dose distributions; for example, dual-field techniques have completely supplanted single-field techniques that use large SSDs [6]. Furthermore, its application and potential modifications in clinical practice at individual radiotherapy units depend on the available equipment and the dimensions of the treatment bunker. Despite its widespread use, several limitations of this technique have been recognized, including the treatment time in a standing position and the combination of six patient positions with irradiation for each to achieve dose uniformity. Despite the implementation of the HDR treatment, which allows for reducing the treatment time [41], the physical strain of standing for the procedure is a notable drawback, particularly for elderly or physically compromised patients [25,31,38,55].

More recent studies have focused on limiting and reducing these critical keys. For this reason, several recent studies have proposed modified versions of the Stanford technique, positioning the patient in a reclined posture during treatment. Each study outlined different setup procedures for the reclined technique, in particular for delivering oblique positions. Specifically, in the studies by Gerbi et al. [25], Lucic et al. [38], and Van der Merwe et al. [27], oblique fields were achieved by rotating the patient and supporting them at the 60° treatment angle using Styrofoam wedges and pillows. In the study by Fuse et al. [30], oblique positions were achieved using suction bags on an inclinable couch. Meanwhile, in the studies by Wu et al. [51], Monzari et al. [56], Ackerson et al. [39], Li et al. [45], and Deufel et al. [37], oblique positions were delivered with the gantry rotated while the patient was positioned on the floor, parallel to the linac waveguide, at a precise lateral distance from the source.

A recent innovation in the Stanford technique is the implementation of a novel shielding approach. In a study by Rahimy et al. [54] a new 3D-printed scalp shielding method was proposed to preserve hair during TSI.

Another alternative to the Stanford technique is the rotational approach, which was examined in a total of eight studies, including mixed studies, where it was compared to the Stanford technique. A key advantage of this approach lies in its potential for improved dose homogeneity and reduced treatment times [14,15,16,51,62,63]. In particular, the rotational technique simplifies the process by reducing the number of dual fields from six treatment positions required in the Stanford method to a single field, thereby decreasing the number of fields and significantly shortening treatment time [14,51]. The studies by Kumar et al. [15] and Piotrowski et al. [14] reported effective dose distributions facilitated by the use of rotational platforms and patient setups designed to maximize radiation exposure while minimizing underexposure to specific areas such as the inner thighs. Techniques such as the ‘swordsman position’ and rotational platforms, as described by Hensley et al. [17], underscore the emphasis on optimizing patient positioning to ensure uniform dose delivery. Despite these advantages, challenges remain. The rotational technique often requires specialized equipment, such as synchronized rotating plates, and careful calibration to ensure platform stability and uniformity of rotation. As highlighted by Falahati et al. [51], patient comfort and safety are significant concerns, with prolonged positioning potentially leading to discomfort and reduced reproducibility. Additionally, the study by Wu et al. [16] proposed a rotational approach for TSI delivery, where patients lie either supine or prone on a rotating board frame. This technique facilitates effective treatment delivery and is well-suited for patients in poor physical condition.

A comparison between the rotational technique and the Stanford technique, as carried out by Ding et al. [58] and Ansari et al. [47], showed that both methods achieve comparable dose uniformity, with the rotational technique being characterized by shorter treatment times and suitability for centers with lower dose rate accelerators. However, practical challenges, including the risk of non-uniform platform rotation, suggest that the technique may not be universally applicable. Regarding clinical outcomes, these articles do not address this aspect in their studies, as they focus on the dosimetric impact, with evaluations conducted on phantoms rather than patients.

Tomotherapy represents a newer documented approach to TSI, offering unique advantages in terms of imaging and delivery precision. The studies by Sarfehnia et al. [39], Haraldsson et al. [49], and Wang et al. [61] demonstrated the importance and utility of daily MVCT imaging to verify the accurate patient setup, enabling real-time corrections to positions prior to treatment delivery. This ensured precise dose alignment, which is critical in minimizing toxicity and optimizing therapeutic outcomes.

However, tomotherapy introduces its own complexities. Immobilization techniques, such as the use of thermoplastic masks and vacuum cushions, are essential for maintaining patient positioning throughout the treatment. The requirement for full neoprene suits as boluses, described in studies by Haraldsson et al. [49] and Wang et al. [61], highlighted the meticulous preparation needed to achieve uniform dose distribution. These setups, while effective, can be resource-intensive and time-consuming.

The studies consistently reported acceptable agreement between calculated and delivered dose distributions, showing benefits for large, convex, cutaneous areas. However, the technique is associated with several limitations, including extended treatment durations—as shown in the study by Haraldsson et al. [49], where the average beam time was 73 min for treatment delivery—and the requirement for daily MVCT imaging. This introduces dosimetric considerations, as daily imaging to verify patient positioning may increase overall exposure. These factors have greatly hindered the development and widespread adoption of this methodology in TSI treatments, potentially resulting in serious side effects such as severe bone marrow suppression. Nonetheless, TSI delivered with electron beams should still be regarded as the standard treatment for primary cutaneous lymphoma, as it effectively covers the entire body surface area [64,65]. Pediatric applications were discussed in four of the articles included [30,35,39,46], underscoring the importance of tailored immobilization devices and the use of anesthesia to ensure safety and efficacy. The Stanford technique has also been described in three studies involving pediatric patients, all of whom were anesthetized during treatment. In two of these studies [30,46], the patients were treated in a lying-down position. In contrast, the study by Bao et al. [35] reported treating the patient in a standing position, using a rotating harness system to achieve the six precise positions for the anesthetized patient. In a single study, treatment with tomotherapy was proposed, and the agreement between the delivered dose and the calculated dose distribution was deemed acceptable [39]. In practice, the treatment was not associated with unacceptable toxicity. However, concerns primarily related to the accuracy of patient positioning during the 7-day treatment period were compounded by the need to verify daily positioning by acquiring one and sometimes two whole-body MVCT scans, which involved additional dosimetric considerations.

### 4.1. Recommendations for Clinical Practice

The TSI technique choice should be tailored to the specific needs and physical capabilities of each patient. An analysis of the current literature on TSI delivery shows that the Stanford technique, with the patient either standing or lying down, as well as the rotation technique, are preferred. The Stanford technique remains the most commonly used method and is recommended for cooperative patients who can maintain an upright posture. By using six different positions, this technique allows coverage of almost all of the patient’s skin with the option of applying a boost to underdosed areas. In addition, novel shielding methods are used to minimize toxicity in certain regions. The weakness of this technique is due to technical issues, such as the necessity of a larger space to obtain an adequate SSD to treat all skin patients and the need for the optimal physical condition of the patient to maintain the position.

For individuals unable to adopt this posture, including pediatric, elderly, or mobility-impaired individuals, supine techniques represent a viable alternative, offering improved comfort and adaptability to compromised clinical conditions.

Innovations such as the HDR technique should be routinely integrated into practice to significantly reduce treatment durations and enhance patient compliance, particularly in frail populations. Rotational techniques, which minimize the number of treatment fields and streamline patient positioning, also present an effective alternative. Notably, the dual rotational approach provides the advantage of reducing SSD requirements while maintaining adequate target coverage, enabling the delivery of TSI even in bunkers with limited dimensions. In light of these advantages of the rotational technique, it is essential to highlight the challenges related to the necessity of implementing specific equipment in clinical practice such as a rotating platform.

For pediatric patients, personalized immobilization systems, combined with anesthesia, are indispensable to ensure safety and efficacy during treatment. An essential requirement for the implementation of TSI in daily clinical practice is the expertise of the multidisciplinary team and the strong collaboration among all the professionals involved. So, TSI is a rare technique and the necessary equipment is not widely available on the market. The preferred technique is often determined by the resources available in each department.

### 4.2. Limitations

The studies analyzed highlighted several constraints that limit the generalizability of the results and their practical applicability. Additionally, the studies included in this review described techniques tailored to their respective radiotherapy centers, taking into account the available equipment and bunker dimensions. Consequently, the positioning and delivery of TSI were customized based on the immobilization devices and infrastructure available. Moreover, many of the included studies rely on small sample sizes or provide insufficient data on the number of treated patients, which undermines the robustness of the available evidence. Finally, the exclusive inclusion of articles published in English could have excluded relevant clinical experiences reported in other languages, further reducing the overall representativeness of the review.

## 5. Conclusions

The Stanford technique has remained the most widely adopted method in TSI over the past four decades, consistently achieving effective results. Its limitations, such as the extended treatment duration and the requirement for complete immobility in an upright position, make it less suitable for elderly or physically frail patients. These challenges have led to increased interest in alternative approaches. Rotational techniques reduce the number of treatment fields, improving efficiency and minimizing irradiation time. Tomotherapy offers advanced immobilization systems and enhanced protection for organs at risk. However, the delivery of high doses to deeper tissues often results in adverse effects, such as bone marrow toxicity, which has significantly hindered the widespread adoption of this technique. These findings underscore the importance of continuous innovation in TSI techniques to improve treatment efficiency, safety, and patient compliance, ensuring their adaptability to diverse clinical contexts.

## Figures and Tables

**Table 1:** Summary of the main findings from the included studies on positioning techniques in TSI.

First Author and Year	State	Sample Size	Technique	Delivery Technique	Energy	Prescribed Dose	Filter or Degrader	SSD	Patient Position	Shielding
Kumar et al. 1982 [15]	36	USA	Rotational	Dual-field technique (gantry angle of ±15° relative to the horizontal axis)	6 MeV	NA	1 cm thick polystyrene screen, 20 cm from the patient	NA	Standing on a rotating platform (5 rpm)	NA
Niroomand-Rad et al., 1986 [24]	NA	USA	Stanford	Six dual-field technique	NA	NA	Dual scattering foils (stainless steel + Al)	3.5 m	Standing on a wooden platform	Eye shield (gold-plated lead, 2.0 to 2.5 mm thick). Uninvolved areas of the body were shielded with lead sheets.
Gerbi et al. 1989 [25]	NA	USA	Stanford	Two electron arc fields (±48° relative to the vertical axis) for each of the six positions	6 MeV	NA	NA	2 m	Reclined on a low couch near the treatment room floor. The oblique fields were achieved by rotating the patient and supporting him using Styrofoam wedges and pillows.	NA
Halberg et al. 1989 [26]	20	USA	Stanford	Six dual-field technique (beams delivered at gantry angles of 250° and 290°)	6 MeV	24 Gy in 6 fractions. Areas not exposed to the beam were boosted with a dose of 12 Gy	NA	3 m	Standing on an elevated platform with an attached supporting frame	NA
Van Der Merwe et al. 1993 [27]	NA	South Africa	Stanford	Six dual-field technique	6 MeV	NA	NA	2 m	Supine or prone on a movable wooden trolley on the floor. The oblique fields were treated with the patient resting on a 60-degree Styrofoam wedge.	NA
Peters et al. 1995 [28]	NA	Canada	Stanford	Six dual-field technique (gantry angle of ±20° relative to the horizontal axis); HDR TSI mode	6 MeV	35 Gy in 20 fractions	An acrylic beam degrader, 6 mm thick, was attached to the front of the stand to reduce electron beam penetration	3.6 m	Standing on an adjustable treatment stand elevated 20 cm above floor level to prevent underdosage at the feet. The stand featured adjustable pegs to provide optimal arm support and included independent turntables for the foot supports for oblique fields.	NA
Weaver et al. 1995 [29]	22	USA	Stanford	Six dual-field technique (gantry angle of ±22/28° relative to the horizontal axis); HDR TSI mode	9 MeV	35/40 Gy	A 1 cm thick acrylic beam spoiler, placed near the patient, was used to scatter the beam	4.1 m	Standing	Lead shields were placed over the contact lens and under the eyelid. Lead shields were placed over the toes to shield the toenails after a dose of 10 Gy.
Earley et al. 1995 [30]	1 pediatric patient (14-month-old child)	USA	Stanford	Twelve-beam irradiation technique (the gantry was set at a 60° angle for an oblique position)	6 MeV	28 Gy in 14 fractions	NA	2 m	Supine and prone on an 80 cm long Plexiglas platform (under anesthesia). For oblique positions, the platform was elevated onto a modified cart and adjusted for the correct distance and orientation.	Lead shields were placed over the fingernails. For the toes, lead strips were coated with wax formed to fit over the nails and nail beds.
Wu et al. 1997 [31]	NA	USA	Stanford	Dual-field technique for AP/PA positions (gantry angle of ±25° relative to the vertical axis). Four fields rotated 60° toward patients for the oblique position. HDR TSI mode	6 MeV	NA	For the AP/PA positions, a 0.6 cm thick acrylic board was positioned 15 cm from the patient. For the oblique position, the acrylic board was placed perpendicular to the beam.	2.13 m for the AP/PA position; 3.3 m for oblique position	Supine and prone positions (laying on the floor).	An eye plaque with a 3 mm lead shield was attached to the surface of the eyeball, functioning as a contact lens. The finger and toenails were covered by a suitable 3 mm lead shielding material.
Antolak et al. 1998 [32]	72	USA	Stanford	Six dual-field technique (gantry angles were set to 113° and 67°); HDR TSI mode	4.29/4.41 MeV at the treatment distance	32 Gy in 16 fractions	A 1.3 cm thick acrylic scatter plate was positioned 25 cm in front of the patient	1.9/2 m	Standing position	NA
Gamble et al. 2003 [33]	NA	Canada	Stanford	Six dual-field technique (gantry angle of ±20° relative to the horizontal axis); HDR TSI mode	6 MeV (5.2 MeV at treatment distance)	NA	A 6 mm thick acrylic sheet was positioned in front of the patient	3.6 m	Standing position	NA
Gamble et al. 2005 [34]	NA	Canada	Stanford	Six dual-field technique (gantry angle of ±20° relative to the horizontal axis); HDR TSI mode	6 MeV (5.2 MeV at treatment distance)	35 Gy in 30 fractions	A 6 mm thick acrylic sheet was positioned in front of the patient	3.6 m	Standing position	NA
Piotrowski et al. 2006 [14]	15	Poland	Rotational	Dual-field technique (gantry angle of ±20° relative to the horizontal axis)	6 MeV	36 Gy in 24 fractions	A 1 cm thick plexiglass board was placed 20 cm from the skin	3 m	Standing position on a rotating platform	Eye shields (lead coated in paraffin) nail shields
Wu et al. 2010 [16]	NA	Taiwan	Rotational	Dual-beam technique for each patient position (gantry angle of ±15.5° from the horizontal); HDR TSI mode	9 MeV	36 Gy in 36 fractions	A 0.6 cm acrylic spoiler was placed 90 cm from the skin to ensure a homogeneous dose	4.5 m	Supine or prone on a homemade rotating board	NA
Bao et al. 2012 [35]	2 pediatric patients (17 months and 12 months)	USA	Stanford	Six dual-field technique (gantry angle of ±20° relative to the horizontal axis); HDR TSI mode	6 MeV	16 Gy in 8 fractions	A 1.2 cm plexiglass scatter plate was placed 25 cm in front of the patient	3.3 m	Standing position (under anesthesia) in the harness system, attached to a frame used for TBI. This harness covered the patient’s torso and was attached to the rotating plate, with four straps looped through carabiner clips.	NA
Platoni et al. 2012 [36]	NA	Greece	Stanford	Six dual-field technique (gantry angle of ±17.5° relative to the horizontal axis); HDR TSI mode	6 MeV	NA	PMMA sheet (203 × 111 cm) of 0.5 cm thickness was placed in front of the patient	3.8 m	Standing position using a suitable immobilization system equipped with two support straps	Eye shields (a pair of goggles filled with Pb of 1.5 cm thickness). Toenails and fingernails (lead layer of 0.4 cm thickness). Genital area (covered with a 2 cm water bag, pasted on top of a 0.4 cm Pb layer). Head (0.4 cm thick lead plate).
Deufel et al. 2013 [37]	NA	USA	Stanford	Three fields for AP and PA positions (angles: 0°, 60°, and 300°). For the oblique position, the gantry was rotated to 300°; HDR TSI mode	6 MeV	NA	A 0.4 cm polycarbonate spoiler was placed 5 cm above the patient’s proximal skin surface	2.31 m for the AP/PA position; 3.12 m for oblique position	In the AP and PA positions, patients were reclined on the floor, oriented head-to-foot perpendicular to the linac waveguide. For the oblique position, the patient was oriented head-to-foot, parallel to the linac waveguide.	Fingernail and eye shields
Luĉić et al. 2013 [38]	NA	Chile	Stanford	Dual vertical beams (0° for field) for each patient position with parallel central axes separated by 80 cm, allowing superior and inferior parts of the body	6 MeV	NA	Custom-made polyester filter and a uniform PMMA degrader plate with a thickness of 8 mm	2 m	Supine or prone on a translational platform	A strip of lead (3 mm thickness) was used to avoid hot spots in the region where the superior and inferior fields overlapped
Sarfehnia et al. 2014 [39]	1	Canada	Tomotherapy	FW of 5 cm, pitch of 0.287, and MF of 2.5. Daily full-body megavoltage imaging using MVCT	6 MV	14 Gy in 7 fractions	NA	NA	Supine position (under anesthesia); immobilized using a Vac-Lok cushion and a thermoplastic mask	Blocks for the brain, thoracic cavity, and abdomen
Fuse et al. 2014 [40]	NA	Japan	Stanford	Six-field technique (comprising four oblique fields and AP and PA gantry orientations)	6 MeV	NA	Two layers of polystyrene, each 2 mm thick, on a 10 mm thick PMMA sheet	1.7–1.8 m	Supine and prone positions on a motorized, inclinable table designed with symmetrical asperities to secure suction bags for patient immobilization. The table moved at a constant speed, synchronized with the linac.	Eyes (lead lenses), nails of fingers and toes (lead plate)
Hensley et al. 2014 [17]	NA	Germany	Rotational	Dual-field technique (gantry angles of 72° and 108°)	6 MeV	NA	6 mm Perspex screen	3.7 m	Standing position on a rotating turntable, with the patient holding a swivel bar to maintain proper posture, keep their arms extended, and provide support while standing	Fingernail, toe, and eye shields
Parida et al. 2013 [41]	25 (14 treated with older technique and 11 treated with HDTSI mode)	India	Stanford	Six dual-field technique (gantry angle of ±15° relative to the horizontal axis); HDR TSI mode	4 MeV	36 Gy in 30 fractions. A boost dose of 10 Gy was delivered to self-shielding regions	Polystyrene screen at a distance of 10 feet. With the implementation of the HDTSI technique, the use of the polystyrene screen was not required.	NA	Standing position on a stationary platform	Eyes and nails shielded with a 3 mm thick lead
Rivers et al. 2016 [42]	NA	USA	Stanford	Six dual-field technique (gantry angles of 248° and 292°); HDR TSI mode	6 MeV	NA	Lucite spoiler accessory	3.77 m	Standing position on an elevated platform	To treat hemibody, a shield consisting of three rectangular layers of plywood with a total thickness of 4.75 cm was used. The plywood layers were clamped on a stand that was positioned 50 cm away from the patient.
Andreozzi et al. 2016 [43]	3	USA	Stanford	Six dual-field technique (gantry angles of 284.5° and 255.5°)	6 MeV	18 Gy in 18 fractions; 36 Gy in 36 fractions	NA	4.41 m	Standing position	NA
Licona et al. 2017 [44]	NA	Mexico	Stanford	Six dual-field technique; HDR TSI mode	6 MeV	NA	A large plastic panel with a thickness of 5 mm was located 20 cm in front of the patient	3 m	Standing position on a wooden platform	NA
Elsayad et al. 2018 [45]	85 (standing, *n* = 77; reclined, *n* = 8)	Germany	Stanford	Six dual-field technique; HDR TSI mode	6–9 MeV	NA	NA	NA	The majority of patients were treated in a standing position, while the remaining 8 patients were treated in a lying position	External eye shields were used during wide-field skin irradiation to protect the cornea and lens, while the nails of the hands and feet were shielded to preserve nail growth
Kron et al. 2018 [46]	1 pediatric patient (2-yr-old patient)	Australia	Stanford	Two sets of six fields (gantry orientations: 0°, 60°, 120°, 180°, 240°, 300°, 360°); HDR TSI mode	6 MeV	18 Gy in 12 fractions. Boost doses of 4 and 6 Gy in six areas (respectively, in 2 and 3 fractions)	1.2 cm thick PMMA block mounted on the gantry	1.24 m	Supine (under anesthesia) on a customized thin window Mylar top (thickness of 0.3 mm) inserted in the treatment couch. After delivery of the first six fields to the upper part of the body, the patient was rotated and the lower half was treated.	Shields were applied to the eyes. Toenail shields were introduced from the third fraction, while fingernail shields were added after the eighth fraction.
Ansari et al. 2018 [47]	NA	Iran	Mixed (Stanford and rotational)	Stanford: six dual-field technique Rotational: dual-field HDR TSI mode	6 MeV	NA	A 0.8 cm PMMA degrader was placed 20 cm in front of the patient	3.5 m	Rotational: standing position on a rotating platform at a speed of 3 rpm	NA
Ibanez-Rosello et al. 2018 [48]	80	Spain	Stanford	Six dual-field technique	6 MeV	NA	A degradative screen (8 mm methacrylate) placed 20 cm in front of the patient	NA	Standing position	NA
Haraldsson et al. 2019 [49]	2	Sweden	Tomotherapy	CT simulation acquired with a slice thickness of 5 mm. FW of 5 cm, pitch of 0.2, and MF of 2.3. Daily MVCT performed.	NA	12 Gy in 6 fractions; 20 Gy in 10 fractions	NA	NA	Supine position with immobilization systems included a large vacuum cushion (VacFix), an individually molded neck rest, and a five-point open-face thermoplastic mask.	Shields for the genitals, eyes, and lips. A hood, gloves, and socks of neoprene.
Diamantopoulos et al. 2019 [50]	NA	Greece	Stanford	Six dual-field technique; HDR TSI mode	6 MeV	NA	A 0.5 cm thick Plexiglas panel	4 m	Standing position using a custom-made immobilization device equipped with adjustable hand grips	NA
Falahati et al. 2019 [51]	NA	Iran	Mixed (Stanford and rotational)	Stanford: six-field technique. Rotational: gantry angle of 90°	6 MeV	NA	A plexiglass filter with a thickness of 5 mm was placed 20 cm in front of the patient	4 m	Stanford: standing position; rotational: standing position on a rotating platform. The platform had a 60 cm diameter, with a height of 81 cm, and rotated at a speed of 3 rpm.	NA
R. Li et al. 2020 [52]	NA	USA	Stanford	Stanford stand-up: six dual-field technique (gantry angles of 251° and 289°). Stanford lay-down: ten electron beams (AP and PA six beams: gantry orientations of 0°, 300°, 60°—oblique four fields: gantry orientation of 300°) HDR TSI mode	6 MeV	NA	A customized Cu (0.25 mm copper disk) scattering filter placed on the interface mount	Stand-up: 3 m; lay down—AP/PA: 1.95 m; oblique: 3.05 m	Stand-up: Standing position on a platform that could rotate at steps of 60°. Lay down: Supine and prone on a platform.	NA
Koken et al. 2021 [10]	26 per year (the yearly average number of patients is 11 in Belgium and 15 in the Netherlands)	Netherlands and Belgium	Stanford	Six fields	4, 6, or 9 MeV	8 × 1.5 Gy on a daily basis; from 7 to 10 × 3 Gy every other day	Lucite diffuser used for 6 and 9 MeV	NA	Standing position (2 institutions) or lying on a stretcher (2 institutions) in prone and supine positions	In three institutions, eyes were shielded using lead goggles, while one institution did not use any shielding (it used the lowest dose scheme)
Tseng et al. 2020 [53]	NA	USA	Stanford	Stanford stand-up: Six dual-field technique (gantry angle of ±15° relative to the horizontal axis). Lay-down: Two vertex fields and four oblique fields (gantry orientation: 60°); HDR TSI mode	6 MeV	NA	Lay-down: 0.25 mm thick copper disc was placed between two 1 mm polycarbonate layers	Stand-up: 3 m	Stand-up standing position on a specific platform. Lay-down: supine and prone	NA
Rahimy et al. 2021 [54]	20 per year	USA	Stanford	Six dual-field technique; HDR TSI mode	9 MeV	NA	Two PMMA spoilers, total thickness of 1.4 cm, roughly 600 cm of air	NA	Standing position	3D-printed scalp shield used to minimize the risk of permanent alopecia
Ackerson et al. 2021 [55]	27 (13 treated in recumbent position and 14 treated in standing position)	USA	Stanford	Lay-down: ten electron beams (AP and PA: six beams with gantry orientations of 0°, 300°, and 60°; oblique: four fields with a gantry orientation of 300°); HDR TSI mode	6 MeV	From 15 to 36 Gy	Lay-down: A customized scattering filter made of 0.025 cm thick copper was placed in the interface mount of the linac	Lay down: AP/PA: 1.95 m; oblique: 3.05 m	Stand-up: Standing position on an elevated platform. Lay down: Supine and prone (five fields each) on a polycarbonate platform with recessed side wheels (body-to-floor distance of 5 cm)	Hands and feet shielded with the stand-up technique
Monzari et al. 2021 [56]	NA	Iran	Stanford	Gantry orientations for AP and PA positions: 0°, 318.5°, 42.5°; Gantry orientation for 4 oblique fields: 291.4°	6 MeV	NA	A beam spoiler made of Plexiglas with a thickness of 5 mm was placed 6.5 cm (for AP/PA fields) or 5 cm (for oblique fields) in front of the patient	AP and PA positions: 2.06 m /0.95 m. Oblique fields: 2.38 m	Supine or prone position	NA
Zhu et al. 2021 [57]	NA	USA	Stanford	Six dual-field technique (gantry angles of 74° and 106°) HDR TSI mode	6 MeV	NA	NA	5 m	Standing position	NA
Ding et al. 2021 [18]	NA	USA	Rotational	Dual-field technique; HDR TSI mode	6 MeV	12 Gy in 12 fractions	An acrylic beam degrader (3 mm or 9 mm thick) was placed 50 cm in front of the patient	3.16 m	Standing position on a rotating platform, which featured a hand-held bar that freely rotated and was anchored to the ceiling	NA
Ding et al. 2022 [58]	NA	USA	Mixed (Stanford and rotational)	Stanford: six-field technique. Stanford and rotational: dual-field technique (for SSD: 3.16 m, 42° difference angle between two beams; for SSD: 5 m, 32° difference angle between two beams); single-field technique (for SSD: 7 m); HDR TSI mode	6 MeV	NA	A 3 mm acrylic scattering plate used for treatment with SSD, 3.16 m and 5 m.	3.16 m, 5 m, 7 m	Stanford: standing position. Rotational: standing position on a rotating platform stand	NA
Baba et al. 2022 [59]	6	India	Stanford	Six dual-field technique	6 MeV	NA	NA	NA	Standing position	NA
Shariff et al. 2022 [60]	NA	Germany	Stanford	Six dual-field technique (289° and 251°); HDR TSI mode	6 MeV	30 Gy in 20 fractions	A planar acrylic scatter plate, 5 mm thick, was placed 50 cm in front of the patient	3.3 m	Standing position	Eye protection, gloves with lead plates to shield fingernails; toenails cover with lead; lead protection for testicles
Wang et al. 2022 [61]	6	China	Tomotherapy	CT simulation acquired with a slice thickness of 5 mm. FW = 5 cm/2.5 cm, pitch = 0.215/0.287, MF = 3/4	NA	24 Gy in 20 fractions	NA	NA	Supine position, immobilized with thermoplastic mask for the head, neck, thorax, and abdomen, and a vacuum cushion for the lower limbs	5 mm diving suit

AP: anterior–posterior, CT: computed tomography, FW: field width, MF: modulation factor, MVCT: megavoltage computed Tomography, NA: not applicable, PA: posterior–anterior, PMMA: polymethyl methacrylate, SSD: source skin distance, TSI: total skin irradiation.

## Supplementary Materials

The following supporting information can be downloaded at: https://www.mdpi.com/article/10.3390/cancers17081276/s1, Table S1: Full search strings for each database.
