# Supplementary material for: Patient Positioning and Treatment Techniques in Total Skin Irradiation: A Scoping Review
Source: Cancers (Basel). 2025 Apr 9;17(8):1276. doi: 10.3390/cancers17081276 (PMC12025804; doi:10.3390/cancers17081276)
Supplement: Supplementary file 1 [file cancers-17-01276-s001.zip › cancers-3511115-supplementary.pdf]

Supplementary material

Table S1. Full search strings for each database.

Database	Search String
PubMed	(position OR set-up OR "set up") AND ("total skin" OR TSI OR TSEBT OR "total skin electron irradiation therapy" OR "Total skin electron therapy" OR "Total skin electron beam therapy" OR tsei)
Embase	('position'/exp OR 'position*' OR 'set-up' OR 'set up' OR 'patient positioning'/exp) AND ('total skin' OR 'TSI' OR 'tsebt' OR 'total skin electron irradiation' OR 'total skin electron therapy'/exp OR 'total skin electron therap*' OR 'total skin electron beam therapy'/exp OR 'total skin electron beam therap*' OR 'tsei')
Scopus	(TITLE-ABS ((position* OR set-up OR "set up") AND ("total skin" OR TSI OR tsebt OR "total skin electron irradiation" OR "Total skin electron therapy" OR "Total skin electron beam" OR tsei))) OR (INDEXTERMS (('position' OR "patient positioning") AND ("total skin electron therapy" OR "total skin electron beam therapy")))
Wos	TOPIC = (position* OR set-up OR "set up") AND ("total skin" OR TSI OR tsebt OR "total skin electron irradiation" OR "Total skin electron therapy" OR "Total skin electron beam" OR tsei
